# Symbionts in waiting: the dynamics of incipient endosymbiont complementation and replacement in minimal bacterial communities of psyllids

**DOI:** 10.1186/s40168-017-0276-4

**Published:** 2017-06-06

**Authors:** Jennifer L. Morrow, Aidan A. G. Hall, Markus Riegler

**Affiliations:** 1 Hawkesbury Institute for the Environment, Western Sydney University, Locked Bag 1797, Penrith, NSW 2751 Australia; 2 Current address: Department of Agriculture and Water Resources, 1 Crewe Place, Rosebery, NSW 2018 Australia

**Keywords:** Core microbiota, Symbiosis, Endosymbiont replacement, *Carsonella*, Psyllid, *Arsenophonus*, *Sodalis*, Enterobacteriaceae, Plant pathogenic bacteria, Insect

## Abstract

**Background:**

Obligate bacterial primary (P-) endosymbionts that are maternally inherited and codiverge with hosts are widespread across insect lineages with nutritionally restricted diets. Secondary (S-) endosymbionts are mostly facultative, but in some hosts, they complement P-endosymbiont function and therefore become obligate. Phylogenetic evidence exists for host switching and replacement of S-endosymbionts. The community dynamics that precede endosymbiont replacement and complementation have been little studied across host species, yet they are fundamental to the evolution of endosymbiosis.

**Results:**

We performed bacterial 16S rRNA gene amplicon sequencing of 25 psyllid species (Hemiptera, Psylloidea) across different developmental stages and ecological niches by focusing on the characterisation of the bacteria other than the universally present P-endosymbiont *Carsonella* (Gammaproteobacteria). Most species harboured only one dominant representative of diverse gammaproteobacterial S-endosymbionts that was consistently detected across all host individuals and populations (*Arsenophonus* in eight species, *Sodalis* or *Sodalis*-like bacteria in four species, unclassified Enterobacteriaceae in eight species). The identity of this dominant obligate S-endosymbiont varied across closely related host species. Unexpectedly, five psyllid species had two or three co-occurring endosymbiont species other than *Carsonella* within all host individuals, including a *Rickettsiella*-like bacterium (Gammaproteobacteria) in one psyllid species. Based on standard and quantitative PCR, all psyllids carried *Carsonella*, at higher titres than their dominant S-endosymbionts. Some psyllids also had Alphaproteobacteria (*Lariskella*, *Rickettsia*, *Wolbachia*) at varying prevalence. Incidence of other bacteria, including known plant pathogens, was low. Ecological niche of gall-forming, lerp-forming and free-living psyllid species did not impact endosymbiont communities. Two flush-feeding psyllid species had population-specific differences, and this was attributable to the higher endosymbiont diversity in native ranges and the absence of some endosymbionts in invasive ranges.

**Conclusions:**

Our data support the hypothesis of strict vertical transmission of minimal core communities of bacteria in psyllids. We also found evidence for S-endosymbiont replacement across closely related psyllid species. Multiple dominant S-endosymbionts present in some host species, including at low titre, constitute potential examples of incipient endosymbiont complementation or replacement. Our multiple comparisons of deep-sequenced minimal insect bacterial communities exposed the dynamics involved in shaping insect endosymbiosis.

**Electronic supplementary material:**

The online version of this article (doi:10.1186/s40168-017-0276-4) contains supplementary material, which is available to authorized users.

## Background

Animals’ diverse interactions with bacterial symbionts can sit anywhere on the scale from parasitism to mutualism, including interactions of invertebrates with maternally inherited intracellular bacterial endosymbionts that provide fitness benefits to hosts. Endosymbiosis can result in genome size reduction of bacterial symbionts and their loss of function [1], requiring complementation or replacement by other endosymbionts [2, 3]. Prior to this, host switches and acquisition of endosymbionts must occur, but these processes have not been studied for entire bacterial communities across many host species. Insects with minimal bacterial communities consisting of a few core species may provide excellent model systems to investigate these symbiont community dynamics.

Insects that feed on plant sap or other nutritionally restricted diets depend on nutrient provisioning by primary (P-) endosymbionts that are localised within specialised host cells and omnipresent in host populations due to the efficient and strictly maternal endosymbiont transmission. This results in co-evolution and can be seen in the phylogenetic congruency of bacterial endosymbionts and hosts [4]. In contrast to obligate P-endosymbionts, many host insects also have facultative associations with other bacteria, secondary (S-) endosymbionts, which may provide fitness benefits to hosts only in a certain ecological or environmental context [5–8]. S-endosymbionts exhibit some key features that are shared with P-endosymbionts: they are intracellular, mostly vertically transmitted (with occasional horizontal transmission), and in some instances fixed in host populations [9]. Furthermore, the genomes of P- and many S-endosymbionts have reduced genome sizes, symptomatic of long-term, obligate endosymbiosis, combined with signs of functional complementation signifying a seemingly stable coexistence of both types of endosymbionts with the host [10, 11]. Endosymbionts are placed under diverse selection pressures, directly from the intracellular host environment [12], and indirectly through environmental and ecological factors such as climate, host plant choice, plant use and plant defences that act on their insect host [13, 14]. Conversely, endosymbiosis drives the evolution of endosymbiont genomes towards accelerated mutation rates and the accumulation of non-lethal mutations [15], gene loss and sometimes lateral gene transfer to the host genome [16]. This may necessitate complementation of P-endosymbiont function by facultative S-endosymbionts that can then become obligate to hosts [3, 10]. It can also result in the loss and replacement of established S-endosymbionts after the acquisition of new endosymbionts by horizontal transmission as evidenced by incongruent phylogenies between S-endosymbionts and hosts [3, 17–21]. Incipient endosymbiont replacement may be detected in hosts containing multiple endosymbionts at varying prevalence within species or varying incidence across related species. Only a few studies have comprehensively characterised entire bacterial communities of hosts both within and across a significant number of species [9, 22], yet such studies are required to detect all present bacteria in hosts, including bacterial symbionts that may evolve from an indifferent transient role (as perhaps seen by a low prevalence or low bacterial titre) to a mutualistic role in their hosts (and they could be referred to as ‘symbionts in waiting’).

Many hemipterans, including plant sap-feeding insects such as aphids, whiteflies and psyllids, have evolved a specialised abdominal tissue structure, the bacteriome, where P- and S-endosymbionts reside. The bacteriome usually consists of bacteriocytes surrounded by a multinucleate syncytium [23]. All species of the hemipteran superfamily *Psylloidea* (in a broad sense referred to as psyllids) harbour the obligate, maternally inherited P-endosymbiont ‘*Candidatus* Carsonella ruddii’ (hereafter *Carsonella*; Gammaproteobacteria) [24]. According to in situ hybridisation studies of the mulberry psyllid, *Anomoneura mori*, and the Asian citrus psyllid, *Diaphorina citri*, *Carsonella* is housed in vesicles within the bacteriocytes, while the S-endosymbionts are localised to the syncytial region [25–27], confirming early microscopic observations of both P- and S-endosymbionts of psyllids [28]. *Carsonella* has an AT-rich and very small genome of approximately 160 kb, with few remaining genes involved in DNA repair, energy metabolism and cell envelope synthesis, as well as having lost complete or partial amino acid synthesis pathways [10, 29]. As a consequence of the minimal genome of *Carsonella* and its reduction in the number of encoded enzymes for amino acid synthesis, it has been predicted that complementing S-endosymbionts may be just as essential to psyllid fitness as the P-endosymbiont [10].

In some hosts, S-endosymbionts show typical signs of obligate mutualism, whereas closely related bacteria found in other host lineages are facultative and do not exhibit extreme gene loss and efficient maternal transmission [30]. For example, whole genome assembly of the Enterobacteriaceae S-endosymbionts of the psyllids *Ctenarytaina eucalypti* and *Heteropsylla cubana* revealed signs of genome reduction, AT bias and metabolic complementarity with the associated *Carsonella* strain [10]. However, unlike *Carsonella* that codiverges with its host, these S-endosymbionts have not strictly codiverged across the entire host phylogeny: while some psyllid lineages appear to show signs of co-evolution with obligate S-endosymbionts [31], there is also evidence for multiple host switching events of obligate S-endosymbionts across several psyllid genera [3, 17], and a few gall-forming psyllid species were reported to exist without any detectable S-endosymbionts [10, 29, 32].

Psyllid species occupy different ecological niches during their nymphal development [33], and this may shape their microbiota. Psyllid nymphs can be free-living, develop within psyllid-induced galls, or develop beneath a ‘lerp’, a cover constructed from solidified psyllid excretions, which offers a protective enclosure that maintains humidity while also restricting nymphs to a particular feeding spot [34]. Lerp building is a predominantly Australian feature common to approximately 50% of Australian psyllid species [35] and could be seen as a transition from the free-living to the gall-forming niche space. The protective and nutritionally enhanced environment of the gall over lerp and free-living niches may reduce endosymbiont reliance of psyllids or influence the type of microbial associations; indeed, studies of gall-forming psyllids have suggested that this may be the reason for the absence of detectable S-endosymbionts in gall-forming *Pachypsylla* spp. and *Cecidotrioza* [10, 32]. However, these are the only genera for which a complete absence of S-endosymbionts has been reported, and another gall-forming psyllid, *Trioza eugeniae* (which forms a pit gall, unlike the enclosed gall of *Pachypsylla* and *Cecidotrioza*), was found to harbour a S-endosymbiont [3]. Furthermore, gall forming is not exclusive to any particular psyllid family, is found in several lineages and has arisen independently: for example, most species of *Glycaspis* (family Aphalaridae) build lerps, but some nymphs of the subgenus *Synglycaspis* form pouch galls on their host plants and species of *Trioza* (family Triozidae) induce galls and pit galls or are free-living [33]. In addition, some psyllid species are considered flush feeders (i.e. they feed on young plant tissue) while others are senescence feeders that favour mature plant tissue [36] and induce early leaf senescence in order to access nutrients that become available due to reallocation within plants [37]. Similar to lerps, apical buds may also provide eggs and nymphs of free-living psyllids with a more enclosed environment that reduces risk of desiccation [38]. It has been found that these different ecological niches impact psyllid life histories and population dynamics [39] and may influence the propensity of different psyllids to acquire and vector plant pathogenic bacteria [40] and, perhaps, other psyllid microbiota.

To investigate the diversity and composition of S-endosymbionts across host lineages, potential signatures of incipient symbiont complementation or replacement and the influence of the nutritional environment in which nymphs develop, we performed high-throughput 16S rRNA gene amplicon sequencing on nymphs and adults of 25 species of 11 genera of four of the eight families of Psylloidea as classified by Burckhardt and Ouvrard [41]. Table 1 describes the specific aims for this study, utilising psyllid species that differ by host plant specialisation (eucalypts and other host plants), geographic locations (including both native and invasive ranges for some species) and ecological niche (free-living, gall-forming and lerp-building species with representation of flush and senescence feeders).

**Table 1:** Experimental aims

1.	Ascertain the entire bacterial diversity and community composition of individual psyllid specimens from 25 psyllid species.
2.	Demonstrate the presence of *Carsonella* in each individual.
3.	Identify the dominant S-endosymbionts that may be complementing the role of *Carsonella* and evolving towards obligate mutualism.
4.	Establish the relative bacterial titre relationships of P- and S-endosymbionts across a subset of seven psyllid species.
5.	Test the previously raised hypothesis that gall-forming species contain a lower diversity of endosymbionts because plant galls may be a more stable nutrient source than unmodified leaf tissue and, more generally, the impact of ecological niche on bacterial community structure.
6.	Test if there are differences in S-endosymbiont incidence and prevalence within psyllid species across locations (including between native and invasive ranges).
7.	Detect potential signatures of incipient symbiont complementation or replacement in terms of loss or acquisition of multiple S-endosymbionts.
8.	Detect potential plant pathogens vectored by psyllid species.

## Methods

### Psyllid collection and characterisation

**Fig. 1:**
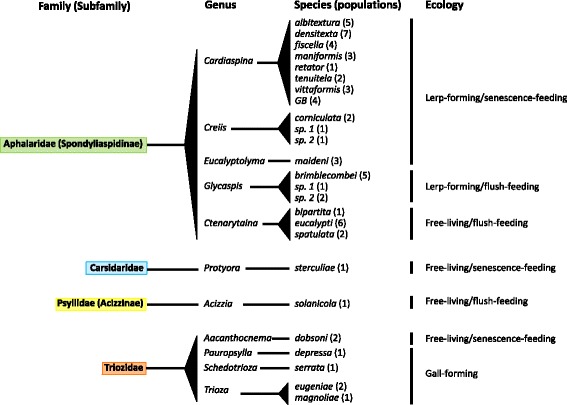
Schematic representation of the psyllid species included in this study. Current taxonomic classification to family level is shown, as are the number of populations collected and the ecological type of each species. Aphalaridae (highlighted in *green*) included in this study are associated with eucalypts; the species from the other families are not associated with eucalypts

Psyllid nymphs and adults were sampled from 25 species of 11 genera of four families within Psylloidea, from host plants mostly in Australia (Fig. 1, Table 2, Additional file 1). Individuals of three species that are invasive outside their Australian-native range were obtained from Portugal, and individuals of one North American species were collected in the USA. One focus of this study was a genus of lerp-building psyllids, *Cardiaspina*, which strictly feed on *Eucalyptus*; some *Cardiaspina* species are significant defoliators and therefore of ecological and economic importance [39, 42]. These were contrasted with other psyllid genera that form distinctive lerps, are free-living or gall-formers (ecology: niche; Table 2), are defined as flush- or senescence feeders [42] (ecology: feeding; Table 2), and developed on the eucalypt genera *Eucalyptus* or *Corymbia* or non-eucalypt trees and shrubs including *Allocasuarina*, *Brachychiton*, *Solanum*, *Ficus*, *Syzygium* and *Persea* (host plant; Table 2). The genus *Cardiaspina* is represented by seven described species and an eighth yet unassigned *Cardiaspina* species responsible for severe defoliation of *Eucalyptus moluccana* (Grey Box) and referred to as GB *Cardiaspina* sp. [43]. The other 10 genera are represented by 13 identified species, in addition to four taxa (two *Glycaspis* sp. and two *Creiis* sp.) that were not classified beyond genus level but were collected from different host plant species and had different DNA barcodes.

**Table 2:** Sampling metadata for the psyllid individuals of 25 species representing four of the eight families within Psylloidea

Family	Species name	Ecology		Location (collection date)	Host plant	No. of individuals		
		Niche	Feeding			Female	Male	Nymph
Aphalaridae	*Cardiaspina albitextura*	Lerp	Senescence	Mudgee; NSW (2015)	*Eucalyptus blakelyi*	3		
				Black Mountain Road carpark, Canberra; ACT (2013)	*Eucalyptus blakelyi*			3
				Black Mountain Peninsula; ACT (2013)	*Eucalyptus blakelyi*	3		
				Bruce Ridge Nature Reserve; ACT (2014)	*Eucalyptus blakelyi*	3		
				Western Sydney U, Hawkesbury; NSW (2012)	*Eucalyptus robusta*			3
	*Cardiaspina densitexta*	Lerp	Senescence	Battunga Road, Echunga; SA (2013)	*Eucalyptus fasciculosa*	3		
				Callington; SA (2013)	*Eucalyptus fasciculosa*			3
				Bunbury Road, Tintinara; SA (2013)	*Eucalyptus fasciculosa*			3
				Tiver’s Road, Spence; SA (2013)	*Eucalyptus fasciculosa*			3
				Hampton Road, Naracoorte; SA (2013)	*Eucalyptus fasciculosa*			3
				Nolan’s Road, Naracoorte; SA (2013)	*Eucalyptus fasciculosa*			3
				Echunga; SA (2013)	*Eucalyptus* sp.			1
	*Cardiaspina fiscella*	Lerp	Senescence	EucFACE, Richmond; NSW (2014)	*Eucalyptus tereticornis*	3		
				Yellowmundee; NSW (2014)	*Eucalyptus tereticornis*			3
				Cronulla; NSW (2014)	*Eucalyptus botryoides*		2	1
				Rotary Park, Southwest Rocks; NSW (2013)	*Eucalyptus botryoides*			3
	*Cardiaspina maniformis*	Lerp	Senescence	Western Sydney U, Hawkesbury; NSW (2014)	*Eucalyptus saligna*	3		
				Macquarie Fields; NSW (2014)	*Eucalyptus tereticornis*	3		
				Yellowmundee; NSW (2014)	*Eucalyptus tereticornis*			1
	*Cardiaspina retator*	Lerp	Senescence	Battunga Road, Echunga; SA (2013)	*Eucalyptus camaldulensis*	1	2	2
	*Cardiaspina tenuitela*	Lerp	Senescence	Mary Lawson Wayside Rest, Finley; NSW (2013)	*Eucalyptus melliodora*			1
				Bruce Ridge Nature Reserve; ACT (2014)	*Eucalyptus melliodora*	3		
	*Cardiaspina vittaformis*	Lerp	Senescence	Doonside; NSW (2012)	*Eucalyptus crebra*			3
				Black Mountain CSIRO, Canberra; ACT (2014)	*Eucalyptus sideroxylon*			3
				Western Sydney; NSW (2012)	*Eucalyptus crebra*	3		
	GB *Cardiaspina* sp*.*	Lerp	Senescence	Rossmore Park; NSW (2013)	*Eucalyptus moluccana*	3		
				Western Sydney U, Hawkesbury; NSW (2014)	*Eucalyptus moluccana*	3		2
				Nurragingy Reserve; NSW (2013)	*Eucalyptus moluccana*	3		
				Dr Charles Mckay Reserve Blacktown; NSW (2013)	*Eucalyptus moluccana*	3		
	*Creiis corniculata*	Lerp	Senescence	EucFACE, Richmond; NSW (2015)	*Eucalyptus tereticornis*	3		
				Glen Helen; NT (2012)	*Eucalyptus camaldulensis*	3		
	*Creiis* sp. 1	Lerp	Senescence	EucFACE, Richmond; NSW (2012)	*Eucalyptus tereticornis*			3
	*Creiis* sp. 2	Lerp	Senescence	Ingleburn; NSW (2013)	*Eucalyptus paniculata*	3		
	*Ctenarytaina bipartita*	Free-living	Flush	Tasman Peninsula; Tasmania (2012)	*Eucalyptus* sp.	3		
	*Ctenarytaina eucalypti*	Free-living	Flush	Sao Pedro do Sul; Portugal (2011)	*Eucalyptus globulus*	3		
				Oliveira de Azemeis; Portugal (2011)	*Eucalyptus globulus*	3		
				Ponte de Lima; Portugal (2011)	*Eucalyptus globulus*	3		
				Gois; Portugal (2011)	*Eucalyptus globulus*	3		
				Tasman Peninsula; Tasmania (2012)	*Eucalyptus globulus*	3		
				Western Sydney U, Hawkesbury; NSW (2012)	*Eucalyptus globulus*	2	1	
	*Ctenarytaina spatulata*	Free-living	Flush	Albergaria-a-Velha; Portugal (2011)	*Eucalyptus globulus*	1		3
				Ponte de Lima; Portugal (2011)	*Eucalyptus globulus*			3
	*Eucalyptolyma maideni*	Lerp	Senescence	Bowen Street Bus Terminal, Adelaide; SA (2013)	*Corymbia maculata*	1		
				Edensor Park; NSW (2012)	*Corymbia citriodora*	2		1
				Western Sydney U, Hawkesbury; NSW (2015)	*Corymbia citriodora*	3		2
	*Glycaspis brimblecombei*	Lerp	Flush	Mulgrave; NSW (2012)	*Eucalyptus saligna*	3		
				East Market Street carpark, Richmond; NSW (2014)	*Eucalyptus saligna*	2	1	
				Coimbra; Portugal (2011)	*Eucalyptus camaldulensis*	2		
				Aveiro; Portugal (2011)	*Eucalyptus camaldulensis*	3		
				EucFACE, Richmond; NSW (2012)	*Eucalyptus tereticornis*			3
	*Glycaspis* sp. 1	Lerp	Flush	Howlong; NSW (2013)	*Eucalyptus* sp.	3		
	*Glycaspis* sp. 2	Lerp	Flush	Mildura; VIC (2015)	*Eucalyptus largiflorens*	2		
				Kinchega; NSW (2015)	*Eucalyptus largiflorens*	3		
				Kinchega; NSW (2015)	*Eucalyptus coolabah*	3		
Carsidaridae	*Protyora sterculiae*	Free-living	Senescence	Western Sydney U, Hawkesbury; NSW (2015)	*Brachychiton populneus*	3		3
Psyllidae	*Acizzia solanicola*	Free-living	Flush	Yellowmundee; NSW (2015)	*Solanum mauritianum*	3		
Triozidae	*Aacanthocnema dobsoni*	Free-living	Senescence	Tasman Peninsula; Tasmania (2012)	*Allocasuarina verticillata*			1
				Bundoora; VIC (2012)	*Allocasuarina verticillata*			3
	*Pauropsylla depressa*	Gall	n/a	Douglas, Townsville; QLD (2014)	*Ficus racemosa*	4		3
	*Schedotrioza serrata*	Gall	n/a	Mount Wilson; NSW (2015)	*Eucalyptus piperita*	3		
	*Trioza eugeniae*	Gall	n/a	Gordon; NSW (2015)	*Syzygium smithii*	3		
		Gall	n/a	Western Sydney U, Hawkesbury; NSW (2015)	*Syzygium smithii*	3		
	*Trioza magnoliae*	Gall	n/a	Kanapaha Plantation Gainesville; Florida (2014)	*Persea borbonia*			3

*n/a* not available

Leaves with psyllid nymphs were collected and placed into zip-lock bags. Nymphs were then either placed directly from leaf surfaces into microcentrifuge tubes with absolute ethanol or allowed to develop into adult males and females and then stored in absolute ethanol. For each species, three to 19 individuals were selected for DNA extraction. Depending on availability, these were adults (females or males) and/or nymphs (fourth or fifth instar) from one to up to seven populations. All specimens were surface sterilised with 4% sodium hypochlorite as described in Morrow et al. [44], and DNA was extracted from whole individuals using DNeasy 96 Blood and Tissue kit (Qiagen) including RNase treatment with 0.4 mg RNase (Sigma). DNA quality and yield was assessed by Nanodrop spectrophotometry, Qubit 2.0 Fluorometry and gel electrophoresis. All samples were initially amplified using general eubacterial 16S rRNA gene primers (63F and 1227R [45, 46]; Additional file 2) to check quality of amplification and with insect mitochondrial *cytochrome b* (CB1 and CB2 [47]) or *cytochrome oxidase I* primers (Pat and Dick [48] and/or LCO1490 and HCO2140 [49]; Additional file 2). cytB and COI amplicons of all individuals were directly sequenced to assess species identity against submissions in the NCBI GenBank database. Because nymphs can potentially be parasitized by parasitoids [50], all nymph sequences were strictly checked for parasitoid cytb or COI sequences as either the dominant or a background sequence within the chromatogram. Nine individuals amplifying parasitoid DNA were eliminated from the study (Additional file 3). COI and cytb sequences were submitted to GenBank, with accession numbers listed in Additional file 1.

Extracts of high molecular weight and amplifiable DNA were submitted to the HIE Next Generation Sequencing Facility for 16S rRNA gene amplification using primers 341F and 805R, which encompass the V3–V4 region of the 16S rRNA gene and produce a fragment of approximately 464 bp, including primers [44]. Library preparation, inputting 7 ng of DNA extract, was performed using the Nextera XT kit and 2 × 300 bp paired-end sequencing on a 384-multiplexed Illumina MiSeq run.

### Data analysis using QIIME

Raw data (fastq format) of 191 individual psyllid libraries were examined using FastQC v0.11.4 (http://www.bioinformatics.babraham.ac.uk/projects/fastqc). Nextera XT libraries are more prone to substitution errors than insertions or deletions, and errors are twice as common in R2 reads compared to R1 reads; thus, trimming at least 10 bp from the 3′ ends of the R1 and R2 reads and merging overlapping reads to correct for errors significantly increases accuracy in these libraries [51]. FastQC demonstrated that the quality of R2 reads of our dataset deteriorated more than R1; therefore, the sequences were trimmed using the trimfq command of seqtk (http://github.com/lh3/seqtk), removing primer sequence and the final 10 bp (−b 17 −e 10) from the forward (R1) reads and and primer and final 90 bp from the R2 reads (−b 21 −e 90). FLASH v1.2.11 (http://ccb.jhu.edu/software/FLASH/) was implemented with default parameters, and this joined the trimmed, paired reads into single sequences with a minimum overlap of 10 bp.

The bacterial community classification and analysis of these samples was performed using QIIME 1.8.0 [52]. Operational taxonomic units (OTUs) were clustered at 97% sequence similarity to the greengenes 13_8-release database using uclust [53] as implemented by the pick_open_reference_otus.py script using default parameters, except that OTUs comprising fewer than three sequences were removed. No negative controls were included in the sequencing dataset; instead, these low read count OTUs were removed during the OTU picking step, so as not to inflate the bacterial diversity due to sequencing error, while still retaining reads of low relative abundance. Representative sequences were chosen as the cluster seed (default parameter). Chimeras were identified using the blast_fragments method [54]. Rarefaction graphs were generated to determine the optimal number of sequences for normalisation of samples. Alpha diversity metrics implemented by ‘alpha_diversity.py’ included observed species, Chao1 (species richness), Shannon and Simpson (diversity) indices and Good’s coverage. Beta diversity was analysed using the phylogenetic distance-based measurement, UniFrac: implementing unweighted UniFrac to assess the relationship between presence and absence of taxa and weighted UniFrac to additionally consider relative taxa abundance. Bray-Curtis similarities were assessed for the presence/absence and relative abundance of OTUs. Statistical tests of alpha and beta diversity comparisons by category (species, genus, ecological niche, whether flush or senescence feeders, and host plant type, i.e. eucalypt versus non-eucalypt) were called by compare_alpha_diversity.py and compare_categories.py.

### 16S rRNA gene sequence from whole genome sequencing approaches

Whole genome sequencing (WGS) of pooled psyllid samples was generated for seven psyllid species: *Cardiaspina albitextura*, *Cardiaspina densitexta*, *Cardiaspina tenuitela*, *Cardiaspina fiscella*, *Cardiaspina maniformis*, GB *Cardiaspina* sp. and *Protyora sterculiae*. For each species, the bacteriomes from 10 adult females were dissected and pooled and the DNA extracted using QiaAmp Mini DNA Extraction kit (Qiagen). In order to maximise the DNA of the endosymbionts within the sample, whole genomic DNA was amplified by multiple displacement with REPLI-G mini kit (Qiagen). The DNA quality and yield was ascertained using Nanodrop spectrophotometry, Qubit 2.0 Fluorometry and gel electrophoresis. The DNA preparations were then submitted for the generation of Truseq DNA PCR-free libraries (350 bp insert, 2 × 101 bp paired end) with 1 μg input DNA. Four libraries (*C. albitextura*, *C. densitexta*, *C. tenuitela* and GB *Cardiaspina* sp.) were run on Illumina Hiseq 1500. The other three libraries were run together with three other libraries on a single lane of Illumina HiSeq2500. The paired fastq output files for each library were imported into CLC Genomics Workbench (ver9.0). Adapters and low-quality reads were removed from the seven libraries by using a trimming function based on the modified Mott algorithm, set to a limit of 0.01. Each library was assembled using default parameters in CLC Workbench. To isolate full-length 16S rRNA gene sequences of *Arsenophonus*, the whole genome of *Arsenophonus nilaparvatae* (NZ_JRLH01000000) was BLAST searched against each assembly to identify matching contigs. Similarly, *C. maniformis* was scanned for sequences matching the *Sodalis glossinidius* str. ‘morsitans’ genome (AP008232) and *P. sterculiae* contigs were identified by BLAST match to the 16S rRNA gene of ‘*Candidatus* Schneideria nysicola’ (hereafter *Schneideria*; HE586114). Complete or near complete 16S rRNA genes were assembled from each library and read mapping performed to ensure accuracy of the sequence and to check for background sequences.

### qPCR

Quantitative PCR was employed to ascertain the relative abundance of the obligate P-endosymbiont *Carsonella* with the dominant S-endosymbionts in 42 individuals of seven *Cardiaspina* species. This was done to verify relative quantification provided by high-throughput amplicon sequencing. The primers 341F and 805R used for high-throughput 16S rRNA gene amplicon sequencing were designed to amplify a wide range of bacteria for microbial community studies and contained no mismatches to the known S-endosymbionts of the *Cardiaspina* psyllids (e.g. *Arsenophonus* and *Sodalis*); however, they contained multiple mismatches in both forward and reverse primers to *Carsonella*, biasing the amplification away from *Carsonella*. Therefore, for quantification relative to *Carsonella* and host, primers were designed using PrimerQuest (IDT) to perfectly match the endosymbiont *atp*A (single copy genes based on published endosymbiont genomes) and host *elongation factor 1 alpha* (*EF1α*) genes for three groups of *Cardiaspina* species, with groupings based on their phylogenetic relationships [3]: (1) *C. densitexta*, *C. tenuitela*, GB *Cardiaspina* sp. and *C. albitextura*; (2) *C. fiscella* and closely related *C. retator*; and (3) *C. maniformis* (Additional file 2). *Carsonella*-and *Sodalis*-specific *atp*A primers were designed for *C. maniformis*, while other *Carsonella* and *Arsenophonus atp*A primers were designed for the other two groups of *Cardiaspina* species (Additional file 2). The primers were designed from consensus sequences derived from whole genome shotgun sequencing, described in the previous section. There was no indication of multiple genome copies or haplotype diversity in these genes. Surprisingly, however, no contig matching the *Arsenophonus atpA* gene was found in the *C. albitextura* whole genome sequence dataset, although *Carsonella atpA* and host *EF1α* genes were both detected with high similarity to *C. densitexta*, *C. tenuitela* and GB *Cardiaspina*. The *C. albitextura* samples were still subject to qPCR with the *Arsenophonus* primers, but no amplification was detected, so for *C. albitextura*, only host *EF1α* and *Carsonella* titres were compared. Primers were designed to amplify products between 97 and 122 bp (Additional file 2).

Efficiency of the primers was tested on a dilution series of genomic DNA extracted from GB *Cardiaspina* sp., *C. fiscella* and *C. maniformis* individuals using the QiaAmp Mini DNA kit (Qiagen). Each template was serially diluted five times (one in 10 dilution) prior to qPCR, to generate primer efficiency curves using Rotor-gene 6000 software (version 2.2.3) (Additional file 4). The qPCR analysis was performed using the Delta Cq (quantification cycle) model without efficiency correction [55] because the efficiency values were within the acceptable range (0.99–1.07).

All primer efficiency tests and experimental qPCR reactions were assembled in duplicate in 100-well rotor discs using a CAS-1200 pipetting robot (Corbett Research). Reaction mix and thermal cycling profile are described in Additional file 2. The melt curve was examined to detect non-target-specific amplicons (none was found) and to ensure little or no primer-dimers were formed. Reference and target gene Cq values were calculated from the arithmetic average of the technical replicate Cq values at the same threshold level. Relative symbiont titre was calculated following normalisation to the host *EF1α* gene copy number using 2^−∆Cq^ [55]. ANOVA and Tukey’s HSD test were performed using the multcomp package [56] in R [57].

### 16S rRNA gene phylogeny

A DNA sequence alignment was produced from 123 sequences derived from amplicon sequencing, whole genome shotgun sequencing and reference sequences downloaded from GenBank. The 56 representative sequences that were selected from the QIIME amplicon sequencing pipeline for the alignment were between 402 and 433 bp in length and included the 50 most abundant sequences plus six *Carsonella* sequences that were detected, but at lower relative abundance due to primer mismatches. Two extra *Carsonella* sequences were extracted from WGS datasets for *C. maniformis* and *P. sterculiae* (430 bp each), as these sequences were not in the representative set of OTUs. Full-length or near full-length 16S rRNA gene sequences of eight S-endosymbionts were extracted from the WGS datasets of *C. albitextura*, *C. densitexta*, *C. fiscella*, *C. maniformis*, *C. tenuitela*, GB C*ardiaspina* sp. (1534-1536 bp) and *P. sterculiae* (two S-endsymbiont sequences 1489 and 1529 bp). Finally, 57 near full-length reference sequences from GenBank were chosen, many of which were published as P- and S-endosymbionts of psyllids, whitefly, aphids and other Hemiptera [10, 17, 24, 25, 31, 58–65].

Sequences were aligned using pynast against the core set of 16S rRNA gene reference sequences within the QIIME pipeline. Partial sequences were included in all phylogenies and coded as missing data (‘?’). The evolutionary model was selected using Bayesian information criterion in MEGA 6.06 [66]. Phylogenetic relationships were estimated using Bayesian inference implemented in MrBayes 3.2.2 [67]. Posterior probabilities were calculated using four independent chains, including one cold, for 20 million generations, sampling every 100 generations, or until convergence was reached (<0.01). The first 25% of trees generated were discarded, and a 50% majority rule consensus tree was returned. FigTree 1.4.0 [68] was used to view the trees.

## Results

### Psyllid samples

Of the specimens initially selected for this study, 93 were nymphs, but nine were eliminated from the study due to parasitisation by parasitoids, detected by amplification and sequencing of the COI gene (Additional file 3), and a further 15 failed the 16S rRNA gene amplicon sequencing. Adult specimens were also COI or cytb barcoded to help the taxonomic grouping of specimens and as reference for future studies. In total, 142 COI sequences and 64 cytb sequences were submitted to GenBank (Additional file 1).

### Psyllid microbiota analysis using 16S rRNA gene amplicon sequencing

A total of 191 individuals from 11 genera, comprising 20 taxa identified to species level and five taxa classified as two *Glycaspis* spp., two *Creiis* spp. and GB *Cardiaspina* sp., were subject to 16S rRNA gene amplicon sequencing and generated a total of 7,076,907 paired-end reads. Following trimming, joining of paired ends and quality filtering of the merged sequence at a phred threshold of >19, sequences were clustered into OTUs at 97% identity and OTUs comprising two or fewer reads were removed. For the remaining OTUs, seven chimeras were identified, but for six psyllid taxa, an excessive proportion of sequences were marked for removal by QIIME due to the identification of a single chimeric OTU common to most or all individuals in each species: for *Aacanthocnema dobsoni*, OTU56 constituting 39–99% of sequence reads obtained from all individual psyllids of this species; for *Creiis* sp., two OTU112 constituting 98.1–98.5%; for *Pauropsylla depressa*, OTU53 constituting 26–97%; for *Eucalyptolyma maideni*, OTU62 constituting 30.4–98.6%; and for *Glycaspis* sp., two OTU38 constituting 38.9–98.7%. One OTU identified as chimeric was found in two out of three individuals of *Glycaspis brimblecombei* from EucFACE, Richmond, but it constituted 93.4 and 95.3% of sequence reads in those individuals. However, these sequences that were flagged as chimeric had NCBI BLAST search hits and aligned (with mismatches) over the full sequence length with published insect endosymbiont 16S rRNA gene sequences. Therefore, we interpreted these OTUs as true, novel bacterial sequences, primarily because when present in an individual, they were highly abundant and they occurred in multiple individuals of the same species. These OTUs were not eliminated from the analysis, and consequently, over all 191 samples, chimera removal was reduced to a maximum of 0.02% of reads per library (Additional file 1). After this, the total number of sequence reads per sample ranged from 970 to 151,787 that were clustered into 4410 OTUs. Representative OTU sequence size ranged from 402 to 433 bp (mean ± std 419.0787 ± 14.1935). Rarefaction curves of observed OTUs per sample pooled by species were generated (Additional file 5) and revealed that read coverage was nearly complete. The Good’s coverage metric indicated normalisation of read number to 970 was sufficient for most samples (Good’s coverage >97.1%, mean 99.2% for samples; Additional file 6) and approached saturation at species level (Good’s coverage >98.3%, mean 99.1% for species). Based on rarefaction to 970 sequences per sample (i.e. the sequence read number of the library with the overall lowest read number), collectively, 1005 OTUs remained, including 729 OTUs that were now only represented by a single read in the entire rarefied dataset (Additional file 7). Alpha diversity metrics showed low species richness with two to 39 detected OTUs (Additional file 6), with no individual psyllid containing more than five OTUs at more than 1% relative abundance.

### Verification of diversity within bacterial clades

Several individuals contained multiple sequence clusters (OTUs) of the same bacterial clade, with one dominant OTU (‘major’ OTU) and one or more minor OTUs present at low relative abundance: this may indicate (1) sequencing error, (2) bioinformatics artefact, (3) multiple 16S rRNA gene copies within individual bacterial genomes where the minor OTU represents the less efficient amplification of a multicopy 16S rRNA gene, or (4) polymorphic bacterial strains. For instance, *Arsenophonus* OTU563559 was the most abundant group of clustered sequences in 85 of the 191 samples (up to 96% of reads), and in those samples consistently co-occurred with a second *Arsenophonus* OTU86606 (up to 5% relative abundance), for which the representative sequence differed by five nucleotides (representing higher identity than the elected 97% clustering cut-off). To validate the OTUs, whole genome sequencing data for the five *Cardiaspina* spp. that harboured *Arsenophonus* were assessed and produced consensus sequences that were somewhat intermediate: they aligned with the major OTU at three SNPs, and the minor OTU at two SNPs, suggesting that some OTU SNPs in the 16S rRNA gene amplicon sequencing set were sequencing errors. Examination of the WGS read mappings at various stringencies for all *Cardiaspina* spp. confirmed the presence of a single isolate with a single 16S rRNA gene sequence variant without any haplotype diversity for three of the five species (*C. tenuitela*, *C. albitextura* and *C. fiscella*). However, two species, GB *Cardiaspina* and *C. densitexta*, which are phylogenetically very close to each other (but different to the other *Cardiaspina* species), had an additional four identical SNPs across the 427-bp region of the 16S rRNA gene. Read mapping validated both this sequence, and the sequence found in the other three species. Only a single *Arsenophonus* contig was produced by the genome assembly algorithm; this was demonstrated by a close BLAST match to the 16S rRNA gene of ‘*Candidatus* Arsenophonus nilaparvatae’, while the next closest matching contig from the assemblies was a single *Carsonella* contig at lower sequence identity. This corroborates the assertion that there is no intraspecies diversity of bacteria within individuals and species and similarly reduces the possibility that these *Arsenophonus* genomes contain more than one 16S rRNA gene sequence variant.

While it is likely that the picking of *Arsenophonus* OTU86606 by QIIME is an analysis artefact in the *Cardiaspina* amplicon libraries (as a consequence of clustering), it was nevertheless found at a relative abundance of 35% in all three *Schedotrioza serrata* individuals in the absence of other *Arsenophonus* OTUs, and this suggests that the OTU represents a valid endosymbiont of this psyllid species.

### Identity of P- and S-endosymbionts belonging to the Gammaproteobacteria

**Fig. 2:**
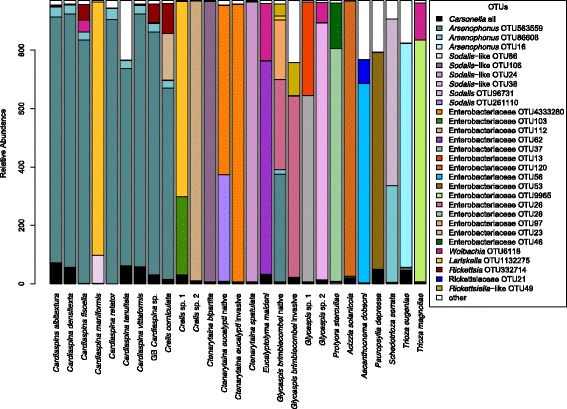
Microbial composition of the dominant OTUs detected in 191 individuals of 25 psyllid species (summarised by species). *Carsonella* (P-endosymbiont), various S-endosymbionts and potential reproductive manipulators were included; others including transient or pathogenic OTUs were grouped into ‘other’. *Glycaspis brimblecombei* and *Ctenarytaina eucalypti* were split into native and invasive samples because for these two species individuals from both native and invasive ranges were available

**Fig. 3:**
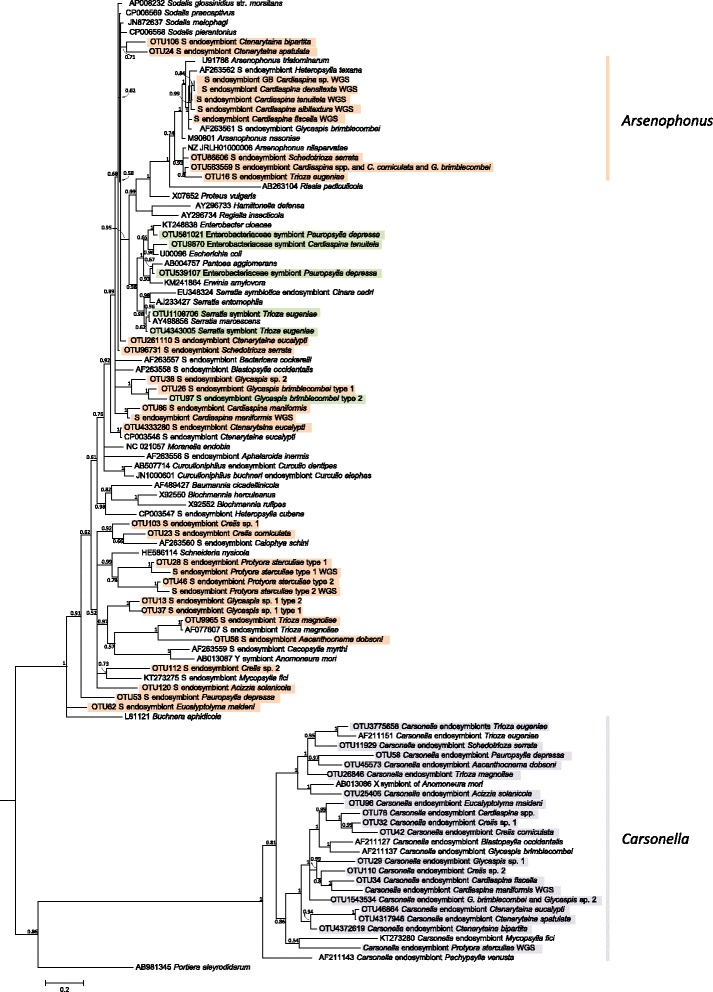
Bayesian phylogenetic tree of 16S rRNA gene sequences, including those acquired from amplicon sequencing of individuals from 25 psyllid species. This tree displays Gammaproteobacteria from the families Halomonadaceae (*Carsonella* sequences from this study highlighted in *purple*) and Enterobacteriaceae which includes the S-endosymbionts that were found in all individuals of a species (or in all individuals of the native range for *C. eucalypti* or *G. brimblecombei*) highlighted in *orange*, or not fixed within a species highlighted in *green*. Posterior probabilities are displayed at the node when >50%. *Scale bar* shows nucleotide substitutions per site. The complete phylogenetic tree with all 16S rRNA gene sequences of this study (including other Gamma- and Alphaproteobacteria) is shown in Additional file 8

Most psyllid species had a single dominant (>80%) S-endosymbiont of the Gammaproteobacteria that was found in all individuals of the same species (Table 3, Fig. 2). *Arsenophonus* and *Sodalis* (or *Sodalis*-like) OTUs were common, with representatives of these bacteria found in nine and five psyllid taxa, respectively, including one species that harboured both. Twelve psyllid species were associated with symbionts unique to their lineage that were identified and classified as endosymbionts of the Enterobacteriaceae family, but it was not possible to confidently place them in any described endosymbiont genus due to the short 16S rRNA gene amplicon or the lack of reference endosymbiont genomes (Fig. 3, Additional file 8). All *G. brimblecombei* individuals also harboured an additional symbiont, a *Rickettsiella*-like OTU (Gammaproteobacteria, Legionellales, Coxiellaceae), at a relative abundance of up to 25%. A fixed S-endosymbiont OTU was found in all populations of a species for all but *G. brimblecombei* and *C. eucalypti* (see the following sections); however, screening included as few as three (up to 19) individuals from each species, and a future wider sampling effort is needed to confirm that these S-endosymbionts are fixed in all populations of a species.

**Table 3:** Bacteria identified by 16S rRNA gene amplicon sequencing

Species name	P-endosymbiont	Potential S-endosymbiont(s)	Facultative endosymbionts (potential reproductive manipulators)	Potentially transient or pathogenic bacteria
Class	Gammaproteobacteria (Halomonadaceae)	Gammaproteobacteria (Enterobacteriaceae + Coxiellaceae)	Alphaproteobacteria + Tenericutes (*)	Gammaproteobacteria + Tenericutes (*) + Firmicutes (#)
		***Arsenophonus*** (>95% BLAST match to NCBI Ref_genomes)		
*Cardiaspina albitextura*	*Carsonella*	OTU563559 (AN = 99%; full-length AN = 99%)		
*Cardiaspina densitexta*	*Carsonella*	OTU563559 (AN = 99%; full-length AN = 99%)		
*Cardiaspina fiscella*	*Carsonella*	OTU563559 (AN = 99%; full-length AN = 99%)	*Wolbachia* (1/12), *Rickettsia* (2/12)	
*Cardiaspina retator*	*Carsonella*	OTU563559 (AN = 99%)		
*Cardiaspina tenuitela*	*Carsonella*	OTU563559 (AN = 99%; full-length AN = 99%)		*Escherichia* (1/4)
*Cardiaspina vittaformis*	*Carsonella*	OTU563559 (AN = 99%)		
GB *Cardiaspina* sp.	*Carsonella*	OTU563559 (AN = 99%; full-length AN = 99%)	*Rickettsia* (1/14)	
*Trioza eugeniae*	*Carsonella*	OTU16 (AN = 97%)		*Serratia* (1/6), *Pseudomonas* (1/6)
		***Sodalis*** **and** ***Sodalis***-**like** (>95% BLAST match to NCBI Ref_genomes)		
*Cardiaspina maniformis*	*Carsonella*	OTU86 (SP = 96%; full-length SP = 97%)	*Lariskella* (7/7)	
*Ctenarytaina bipartita*	*Carsonella*	OTU106 (SP = 96%)		
*Ctenarytaina spatulata*	*Carsonella*	OTU24 (SG = 96%)		
*Glycaspis* sp. 2	*Carsonella*	OTU38 (SG = 95%)	*Wolbachia* (1/8)	
		**Unclassified Enterobacteriaceae**		
*Creiis* sp. 1	*Carsonella*	OTU103 (SG = 93%)	*Lariskella* (3/3)	
*Creiis* sp. 2	*Carsonella*	OTU112 (BP = 90%)		
*Eucalyptolyma maideni*	*Carsonella*	OTU62 (ME = 93%)	*Wolbachia* (4/9)	
*Glycaspis* sp. 1	*Carsonella*	OTU37 (ME = 91%) + OTU13 (ME = 92%)		
*Acizzia solanicola*	*Carsonella*	OTU120 (CE = 92%)		
*Aacanthocnema dobsoni*	*Carsonella*	OTU56 (WG = 87%)	*Rickettsia* (1/4)	*Phytoplasma* (2/4)*
*Pauropsylla depressa*	*Carsonella*	OTU53 (ME = 92%)		*Erwinia* (2/7), *Pseudomonas* (2/7), *Enterobacter* (1/7)
*Trioza magnoliae*	*Carsonella*	OTU9965 (BA = 89%)	*Wolbachia* (3/3)	
		**Multiple S-endosymbionts**		
*Creiis corniculata*	*Carsonella*	OTU563559 (*Arsenophonus* AN = 99%) + OTU23 (CE = 93%)	*Rickettsia* (1/6)	
*Protyora sterculiae*	*Carsonella*	OTU28 (*Schneideria*-like CE = 92%; full-length CE = 90%) + OTU46 (*Schneideria*-like BP = 91%; full-length BP = 91%)		
*Schedotrioza serrata*	*Carsonella*	OTU96731 (*Sodalis* SP = 99%) + OTU86606 (*Arsenophonus* AN = 99%)		
*Ctenarytaina eucalypti*				
Native (Tasmania)	*Carsonella*	OTU4333280 (CE = 100%) + OTU261110 (*Sodalis* SP = 97%)		
Invasive (Portugal)	*Carsonella*	OTU4333280 (CE = 100%)		
*Glycaspis brimblecombei*				
Native (Mulgrave, NSW)	*Carsonella*	OTU26 (SG = 93%) + OTU563559 (AN = 99%) + OTU49 (*Rickettsiella*-like DM = 92%)		
Native (Richmond, NSW)	*Carsonella*	OTU26 (SG = 93%) + OTU563559 (AN = 99%) + OTU49 (*Rickettsiella*-like DM = 92%)	*Spiroplasma* (1/3)*	
Native (EucFACE, NSW)	*Carsonella*	OTU26 (SG = 93%) + OTU563559 (AN = 99%) + OTU49 (*Rickettsiella*-like DM = 92%)	*Lariskella* (1/1)	
Native (EucFACE, NSW)	*Carsonella*	OTU97 (SG = 92%) + OTU49 (*Rickettsiella*-like DM = 92%)		
Invasive (Portugal)	*Carsonella*	OTU26 (SG = 93%) + OTU49 (*Rickettsiella*-like DM = 92%)		*Staphylococcus* (2/5)#

All psyllids harboured *Carsonella* and fixed S-endosymbionts, and 17% had facultative endosymbionts or transient/potentially pathogenic bacteria. All psyllids harboured abundant S-endosymbionts, many of which were difficult to classify to genus due to the short region of 16S rRNA gene sequenced and the similarity of the sequenced region across several insect endosymbiont taxa. The S-endosymbiont OTUs were grouped into *Arsenophonus* or *Sodalis*-like (based on >95% BLAST matches against the NCBI reference genomes database); matches lower than 95% were grouped as ‘unclassified Enterobacteriaceae’ with the closest reference genome BLAST match. *NB* In the last two columns, numbers in parentheses indicate the fraction of the total individuals that harboured the bacteria

Genome abbreviations: *Arsenophonus nilaparvatae* NZ_JRLH01000006 (AN), *Sodalis glossinidius* ‘morsitans’ NC_007712.1 (SG), *Sodalis praecaptivus* strain HS1 NC_007712.1 (SP), *S-endosymbiont of Ctenarytaina eucalypti* NC_018419.1 (CE), *Candidatus Blochmannia pennsylvanicus* str. BPEN NC_007292.1 (BP), *Candidatus Moranella endobia* PCVAL NC_021057.1 (ME), *Buchnera aphidicola (Cinara tujafilina)* NC_015662.1 (BA), *Wigglesworthia glossinidia* NC_016893 (WG), *Diplorickettsia massiliensis* NZ_AJGC01000002.1 (DM)

In addition, 17 OTUs were classified as *Carsonella*, the obligate P-endosymbiont of psyllids. *Carsonella* sequence reads in individuals ranged between 0 and 29%. There were 10 samples that did not produce any *Carsonella* reads in the full dataset: all six *P. sterculiae*, two *C. fiscella* and two *C. maniformis* individuals. The *C. fiscella* and *C. manifomis* were included in the qPCR experiment, and the presence of *Carsonella* was confirmed in each individual in high but varying titres (see the ‘qPCR’ section); *P. sterculiae* individuals are known to harbour *Carsonella* [3] but were also subjected to *Carsonella*-specific PCR which confirmed the presence of *Carsonella* in all individuals.

### Relative quantification of P- and S-endosymbiont titres by qPCR

**Fig. 4:**
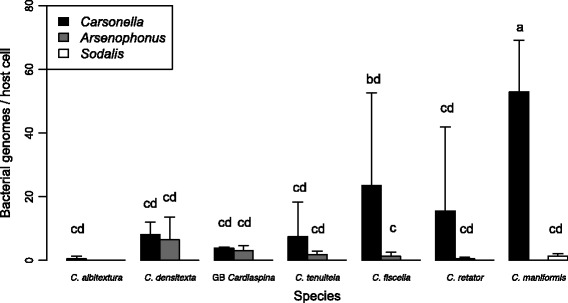
Relative titres of P- and S-endosymbionts in *Cardiaspina* spp. determined by qPCR. Cq values for *Carsonella*, *Arsenophonus* and *Sodalis* were normalised to the EF1a host gene (see Additional file 8), and species means of endosymbiont copy number were plotted. Tukey’s post hoc tests were performed and the results displayed over each bar, demonstrating no significant difference between the titres of the P- and S-endosymbionts within and between four species, but significantly higher *Carsonella* titres for *C. fiscella* and *C. maniformis* than the other species*. Cardiaspina albitextura* was included to display relative *Carsonella* titre only; while *Arsenophonus* was present in each individual of *C. albitextura* based on amplicon sequencing data, it was not detected using the *atp*A qPCR primer set

According to qPCR, *Carsonella* was much more abundant than the 16S rRNA gene amplicon sequencing analysis suggested (Additional file 9; Fig. 4). Most individuals had higher titres for *Carsonella* than *Arsenophonus* (21 out of 30 individuals, 1.1- to 383-fold higher titre), and *Carsonella* titre was 16- to 261-fold higher than *Sodalis* in the seven *C. maniformis* individuals. *Carsonella* was relatively more abundant than host cells (in 32 out of 42 individuals), notwithstanding the extraction of DNA was from whole psyllids, and *Carsonella* is restricted to the abdominal bacteriome. The titre of *Carsonella* in *C. fiscella* samples 124 and 125 was five- to eightfold higher than host cells, and 19- to 22-fold higher than *Arsenophonus*, contrary to the absence of *Carsonella* reads in the MiSeq amplicon dataset. *Carsonella* was on average 53 times higher than *C. maniformis* host cells, and while *Sodalis* was detected at much lower titres (0.1 to 2.5× relative to host cells), it was in the same range as *Arsenophonus* in the other *Cardiaspina* species.

The titre of the endosymbionts was highly variable across individuals of the same species (Fig. 4) and did not vary according to developmental stage or sex (chi-square test df = 2, *p* = 0.333). *Carsonella* titre in C. *maniformis* was significantly higher than the *Carsonella* titre in all other *Cardiaspina* spp. and may correlate to the association with *Sodalis* in C. *maniformis* (Fig. 4).

Furthermore, the primer development for the qPCR assay revealed that the *Arsenophonous* S-endosymbiont of *C. albitextura* lacked *atpA* which confirmed *Arsenophonus* strain diversity across the different *Cardiaspina* species [3] and requires further investigation with regard to the significance of this loss for genome function.

### Co-occurrence of multiple S-endosymbiont genera in some psyllid species

**Fig. 5:**
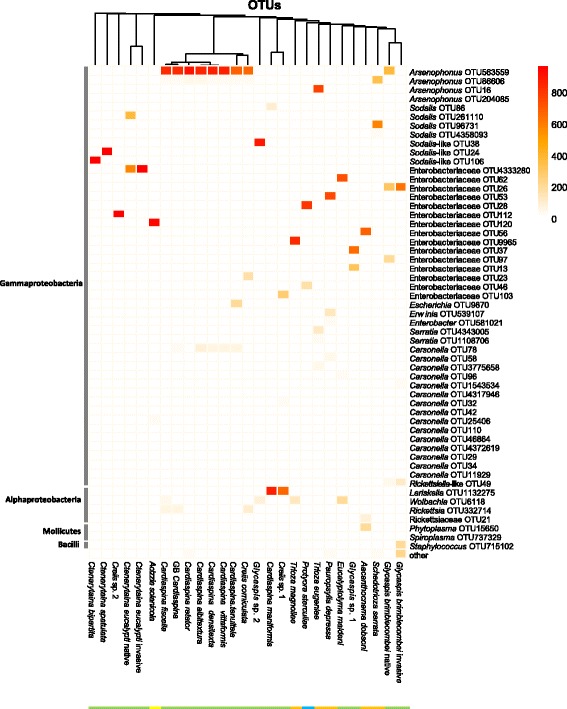
Heatmap showing relative abundance >1% of OTUs labeled to genus or family level in each species. *Each row* represents a microbial OTU, and *colour shading* indicates the relative abundance according to the *inset* legend on the *top right. Columns* represent each psyllid species (*C. eucalypti* and *G. brimblecombei* samples were also divided into specimens of native and invasive populations) and were ordered by Euclidean hierarchical clustering. Class level designations for the microbial OTUs are presented on the *left* of the figure. Psyllid families are indicated by the *coloured bar*: Aphalaridae (*green*), Carsidaridae (*blue*), Psyllidae (*yellow*) and Triozidae (*orange*)

The bacterial communities of several psyllid species contained multiple S-endosymbionts at high relative abundance (Table 3, Fig. 5). All *S. serrata* individuals harboured both *Arsenophonus* and *Sodalis* OTUs (10.3% divergence in 427 bp) and clearly demonstrated coinfection by two distinct bacterial lineages at an average relative abundance of 34 and 59%, respectively. Similarly, *Creiis corniculata* possessed *Arsenophonus* (68% of reads) and a distinct Enterobacteriaceae OTU (16% of reads) with a 12.9% divergence in their 16S rRNA gene sequence. In *P. sterculiae*, two distinct S-endosymbionts (9.6% divergence) classified to Enterobacteriaceae were detected, and these OTUs were present in each individual at an average of approximately 82 and 16% relative abundance. The presence of these diverged sequences in *P. sterculiae* was corroborated by the WGS dataset: both sequences were detected by mapping reads to the two OTU sequences at high stringency, and the differential abundance based on sequence coverage (ratio 49:1) in the WGS dataset demonstrated that these were not multiple intra-genomic 16S rRNA gene copies. Hence, two independent methods validated the presence of two distinct Enterobacteriaceae S-endosymbionts in *P. sterculiae*, one at high and one at low titre.

In *Glycaspis* sp. 1, two Enterobacteriaceae OTUs were detected in each individual but they differed by only 3% and may therefore be haplotypes of a single bacterial species that had diverged after acquisition by the host insect, as opposed to different bacterial lineages that had been independently acquired (such as seen in *P. sterculiae*).

Low count reads need to be treated with caution; however, our detection of bacterial lineages at low relative abundance in all individuals of some but not all host species (therefore potential fixation in some species) needs noting. Although *T. eugeniae* possessed a distinct *Arsenophonus* sequence, each individual also harboured low levels (mean proportion of 0.9%) of the dominant *Arsenophonus* found in most individuals of the *Cardiaspina* genus. Each individual of *P. sterculiae*, *Acizzia solanicola* and *Trioza magnoliae* also harboured low levels of this *Arsenophonus* sequence (mean proportion of 0.3–0.5%), and this is in the absence of a relatively abundant *Arsenophonus* OTU dominating the bacterial community in each of these species. However, *Arsenophonus* sequences were not detected in the *P. sterculiae* WGS data set.

Specimens of *G. brimblecombei* were collected from both the native Australian range and from Portugal where it is invasive. All individuals had a *Rickettsiella*-like bacterium. In *G. brimblecombei* from Australia, six out of nine individuals also carried both *Arsenophonus* and OTU26, a currently unclassified Enterobacteriaceae S-endosymbiont (13.1% divergence separating the two S-endosymbionts). The remaining three specimens carried a single Enterobacteriaceae endosymbiont—the common OTU26 in one and a related OTU97 (3.4% diverged) in the other two individuals. In the Portuguese samples, only the common OTU26 endosymbiont was found. Similar differences in invasive versus native populations were detected in *C. eucalypti*, where the previously published S-endosymbiont of *C. eucalypti* [10] co-occurred with a less abundant *Sodalis*-like bacterium (that was differentiated by 4.7% nucleotide diversity) in all specimens from the native Australian range, but only the more abundant endosymbiont was detected in the Portuguese samples. For all other species examined in this study, all replicated populations (from one to seven populations per species) harboured fixed S-endosymbionts.

### Endosymbionts belonging to the Alphaproteobacteria and other bacterial classes

Alphaproteobacterial 16S rRNA gene sequences were found in psyllids that were not dominated by Gammaproteobacteria (but still contained Gammaproteobacteria as S-endosymbionts). The OTUs included *Wolbachia* (Rickettsiales, Alphaproteobacteria) which was detected at high relative abundance (11–66%) in some individuals of *C. fiscella*, *E. maideni* and *Glycaspis* sp. 2 and in all *T. magnoliae*, as well as at very low relative abundance below 1% and low prevalence in 14 individuals of nine species (Additional file 7). Two *Rickettsia* (Rickettsiales) OTUs that displayed 96.8 and 99.3% identity to the *Rickettsia* endosymbiont of the pea aphid (GenBank KP710438) were at high relative abundance in one of the four *A. dobsoni* individuals (OTU21), one of the six *C. corniculata*, one of the 14 GB *Cardiaspina* sp. and two of the 12 *C. fiscella* (OTU332714). *Lariskella* (Rickettsiales) was dominant and fixed in *Creiis* sp. 1 and *C. maniformis. Spiroplasma* (Tenericutes) was detected in a single *G. brimblecombei* individual, but at a modest 2.8% relative abundance. The Alphaproteobacteria at high relative abundance always co-occurred with gammaproteobacterial S-endosymbionts in all of these psyllids, albeit with the gammaproteobacterial S-endosymbiont at a lower relative abundance in each individual (Fig. 5).

### Transient bacteria and potential plant pathogens

High relative abundance of *Phytoplasma* sp. was detected in two out of the four *A. dobsoni* individuals (20 and 59% of reads) collected from *Allocasuarina verticillata*; these were isolates with 99% similarity to a *Phytoplasma* found in *Allocassuarina muelleriana* [69], where it causes yellowing of the foliage. ‘*Candidatus* Liberibacter spp.’, causal agents of plant diseases including Huanglongbing in citrus and zebra chip disease in potatoes are vectored by psyllids. However, no *Liberibacter* was detected in any of the samples examined here. Other bacteria that were detected in some psyllid individuals at high relative abundance included OTUs that were similar to *Erwinia* and *Pseudomonas.*

### Bacterial community diversity

Psyllid bacterial communities were generally low in species richness and dominated by few relatively abundant species (Additional files 6 and 7). Based on the rarefaction analysis, for the number of OTUs (observed_species) detected in each psyllid species, it appeared that the mean number of OTUs found in the gall-forming *S. serrata* was highest and more than twice as high as in any other psyllid species (Additional file 5). Furthermore, the next psyllid species that were ranked highest with regard to the number of OTUs included the other three triozid gall-forming species (*T. eugeniae*, *T. magnoliae* and *P. depressa*) and the flush-feeder *G. brimblecombei*, however, there was no overall statistical support for this.

Comparison of the alpha diversity metrics Shannon, Simpson and the number of observed species (compare_alpha_diversity.py) using non-parametric statistics showed that free-living psyllids harboured the least diverse bacterial communities compared to lerp builders and gall formers. Both lerp (*t*-stat = 3.23, *p* = 0.012)- and gall formers (*t*-stat = 3.02, *p* = 0.012) had significantly higher Shannon diversity than free-living psyllids, and gall-forming psyllids were significantly higher than free-living psyllids for the Simpson metric (*t*-stat = 2.36, *p* = 0.063). Senescence feeders had higher Shannon diversity (*t*-stat = 2.98, *p* = 0.018) and observed species (*t*-stat = 4.55, *p* = 0.003) than flush feeders, but no difference in the Simpson metric. There were no significant differences in Shannon, Simpson or observed species metrics when samples were grouped by genus or species. Too few males were sampled for statistical rigour, but a comparison of nymphs versus adult females for species with adequate sampling (i.e. *C. albitextura*, *C. fiscella*, *P. depressa* and *P. sterculiae*) also returned no significant difference for any alpha diversity metric. These results seemed to be driven by the higher incidence of facultative Alphaproteobacteria (e.g. *Wolbachia*, *Rickettsia*, *Lariskella*) and transient (possibly gut associated) or pathogenic bacteria detected in gall- and lerp-forming psyllids (Table 3).

**Fig. 6:**
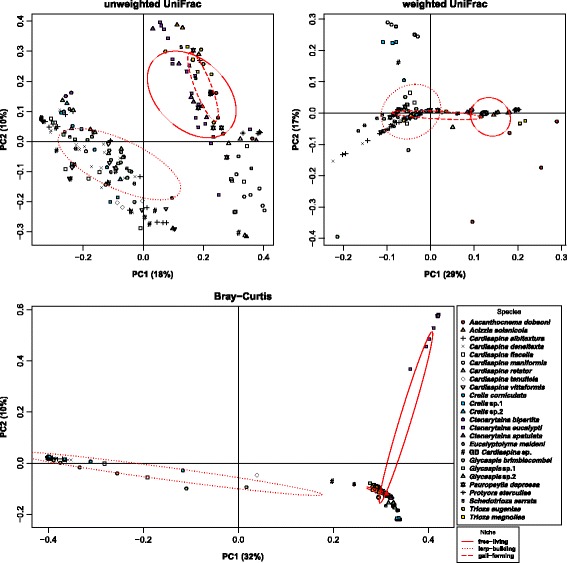
Principal coordinates analysis (PCoA) of three distance metrics: unweighted UniFrac, weighted UniFrac and Bray-Curtis, based on OTUs calculated at 97% similarity. Microbial communities of 191 psyllid individuals of 25 species are represented. *Axes labels* denote the percentage of variation that is contained in each principal coordinate; *ellipses* represent the standard deviation of the cluster centred on each ecological niche

Beta diversity comparisons of weighted and unweighted UniFrac and Bray-Curtis dissimilarity metrics showed significant differences when categorised at all levels: species, genus, niche, feeding type and host plant (Additional file 10). However, species identity governed the minimal microbial composition (Fig. 5), and the unweighted UniFrac PCoA plot (Fig. 6) displayed the loose clustering of the lerp-forming species (mostly *Cardiaspina*, *Glycaspis* and *Creiis* species harbouring genetically similar *Arsenophonus* OTUs), as distinct from the loose cluster of free-living and gall-forming species, many of which harboured *Sodalis*-like OTUs. This clustering was more pronounced in the weighted UniFrac when abundance was included, due to the dominance of the S-endosymbiont read count. The Bray-Curtis PCoA plot contrasted with UniFrac to differentiate the OTUs as separate entities irrespective of phylogenetic similarity, hence the tight clustering of the multiple *Cardiaspina* species which carried the same single dominant OTU and the spread of species that either (1) carried relatively abundant facultative endosymbionts, (2) harboured relatively abundant transient or opportunistic bacteria or (3) harboured multiple S-endosymbionts at high relative abundance (Fig. 6).

## Discussion

We analysed the complete bacterial diversity and community composition of 191 individuals of 25 diverse psyllid species, including seven species for which bacterial associates were characterised for the first time. This is the most comprehensive analysis of psyllid-associated bacterial diversity and composition across species so far, and it revealed for each psyllid species a minimal community of core microbiota consisting of only a few bacteria, including *Carsonella* as universal P-endosymbiont and at least one S-endosymbiont belonging to Gammaproteobacteria. P- and S-endosymbionts were found in all individuals of psyllid species, confirming strict maternal inheritance of this minimal bacterial core community. The 16S rRNA gene amplicon sequencing analysis detected for most individuals the presence of at least one dominant Enterobacteriaceae S-endosymbiont taxon that was fixed across all tested individuals of host species, and supported the hypothesis that complementation by another endosymbiont may have led to co-dependencies between psyllid P- and S-endosymbionts [3, 17]. The species identity of this presumably complementing S-endosymbiont varied across host taxa, such that closely related host taxa (e.g. within psyllid genera) had different dominant S-endosymbionts, thereby supporting the hypotheses of host switching and replacement of S-endosymbionts in psyllids. Furthermore, our study of psyllid microbiota provided an unexpectedly rich source of new and undescribed endosymbiont diversity, with unclassified S-endosymbionts (all belonging to Enterobacteriaceae) in 12 of the 25 psyllid species. Interestingly, five psyllid species possessed two or three distinct and highly abundant S-endosymbiont taxa, and this may constitute examples of ongoing endosymbiont complementation or replacement as it is expected to occur based on earlier cophylogenetic analyses that demonstrated host switches for S-endosymbionts of psyllids [3, 17]. For other psyllid species, besides the dominant S-endosymbiont, we detected low-frequency reads of other S-endosymbionts in all individuals and these ‘symbionts in waiting’ may form the basis of future endosymbiont complementation or replacements.

### Psyllids have ubiquitous and novel S-endosymbionts

The overall low diversity of bacteria found within 25 psyllid species aligns with previous studies of other hemipterans including studies of a number of psyllid species [22, 70]. Dominant bacterial clades found in each psyllid species were related to common insect endosymbionts localised to bacteriomes, including *Arsenophonus* [26], *Sodalis* [71], the Enterobacteriaceae S-endosymbiont of *A. mori* [25] and *Schneideria*, the bacteriome-associated P-endosymbiont of stinkbugs [72]. All *Cardiaspina* and *Ctenarytaina* species harboured S-endosymbionts classified to either *Arsenophonus*, *Sodalis* or *Sodalis-like* bacteria; however, in several psyllid species, we discovered new bacterial endosymbiont taxa for which there are no genomic references in databases. Many fell into diverse clusters within Enterobacteriaceae that remained phylogenetically unresolved due to the short 16S rRNA gene amplicon used for most species, and future work should involve comprehensive analysis of these undescribed bacteria using full genomes or multilocus characterisation. Also particularly noteworthy is the finding of a *Rickettsiella*-like bacterium in all *G. brimblecombei*. Previously a *Rickettsiella*-like bacterium has been described from *Cecidotrioza sozanica* [32], and *Rickettsiella* has been found to impact body colour of the aphid *Acyrthosiphon pisum* [73]. This may also play a role in *G. brimblecombei* which also displayed green/red colour polymorphism (MR, personal observation), and this warrants further investigation.

The potential for abundant S-endosymbionts to evolve to obligate mutualism is supported by bacterial community analysis by UniFrac-based PCoA which demonstrated that psyllid phylogenetic relationships were the most important determining factors in clustering the microbiota. Certain characteristics of obligate bacterial associations (such as vertical transmission, high titre, 100% prevalence in all populations of a species) are strongly supported by the data; however, facultative endosymbionts (those that are not strictly required by the host, e.g. *Wolbachia*) may also embody these characteristics. Vertical transmission of *Arsenophonus* in three *Cardiaspina* species has previously been demonstrated by detection of this endosymbiont within surface-sterilised eggs [3]. At higher taxonomic levels, it has also been found that some but not all species within psyllid genera (or within a family, for example *A. dobsoni* and *T. magnoliae* of Triozidae) tended to be associated with related isolates of the same S-endosymbiont lineage supporting a combination of repeated host switches into the same host lineage (host tracking) and codivergence [3, 31].

### Influence of ecological niche and host plant use on microbiota

It has previously been reported that some gall-forming psyllid species were depauperate of S-endosymbionts and this led to the hypothesis that S-endosymbionts may not be essential to gall-forming psyllids, perhaps due to the more nutrient-rich habitat of galls when compared to lerp and free-living niches [32]. However, we found 100% presence of gammaproteobacterial S-endosymbionts; *Wolbachia* was also fixed in *T. magnoliae*. Alpha diversity metrics also contradicted the hypothesis of depauperate S-endosymbionts in gall formers, with lower diversity found in lerp-forming and free-living psyllids. Bacterial community comparisons across psyllid species with not only different ecological niches but also different feeding habit (flush or senescence) and host plant (eucalypt and non-eucalypt) did not demonstrate a clear effect of any of these factors due to the strong effect of psyllid species on microbial composition. Inclusion of species within a genus that exhibit different characteristics (such as gall-forming species of the *Glycaspis* subfamily *Synglycaspis* to contrast with the more common lerp-building species or a larger sampling of a given psyllid species that has multiple host tree associations) will be required to tease out these effects on bacterial composition and diversity.

### Endosymbiont loss in invasive ranges

A striking difference in S-endosymbiont incidence was found between native Australian and invasive populations of *G. brimblecombei* and *C. eucalypti*. In both cases, two relatively abundant S-endosymbionts were detected in the native range, but only a single dominant S-endosymbiont was detected in the invasive samples from Portugal. This is also confirmed by a previous report for *G. brimblecombei* in California where only *Arsenophonus* and no other endosymbiont was found [17], at an overall prevalence of 40%, with variability ranging from 0 to 75% across Californian populations [5], and whole endosymbiont genome sequencing analysis of invasive *C. eucalypti* in California has also revealed the presence of a single S-endosymbiont [10] without the presence of the additional endosymbiont that we found in Australian specimens of this species. Furthermore, *D. citri* specimens from Florida were bereft of the *Arsenophonus* strain detected in Asian samples from where this psyllid originates [26, 74]. The examples of *G. brimblecombei* and *C. eucalypti* suggest a dynamic endosymbiont community, with the potential loss of microbial diversity in the invasive ranges, with impacts on host insects that may rely on endosymbiont function for nutrition and development. Loss of some but not all endosymbionts has previously been reported for invasive host populations of a thrips (e.g. loss of *Wolbachia* but not *Cardinium* in invasive *Pezothrips kellyanus*), possibly due to incomplete transmission, stochastic effects or selection pressures that vary throughout host ranges [75].

### Symbiont replacement

Previous studies have found *Carsonella* in all psyllid species [3, 24]. In our study, each psyllid also harboured *Carsonella*, and qPCR results showed that *Carsonella* was more abundant than S-endosymbionts in individuals of *Cardiaspina*, although at extremely variable titres*.* The dominance of *Arsenophonus* or *Sodalis* bacteria in all *Cardiaspina* individuals and species (although at titres generally lower than *Carsonella*) also supported the notion that association with S-endosymbionts can be viewed as obligate symbiosis for psyllids. However, some psyllid species typically had multiple S-endosymbionts in all individuals (some at high and others at low relative abundance), and this may be indicative of ongoing endosymbiont complementation or replacement (e.g. [3]). The characterisation of the ecological processes leading to endosymbiont complementation or replacement, particularly in their early stages (i.e. incipient endosymbiont complementation or replacement), will be critical to understanding the evolution of endosymbiosis and the frequency of endosymbiont complementation and replacement across host species. Our multispecies study has demonstrated potentially new endosymbiont combinations within and across related psyllid species, and this will highlight the chronology of changes that may occur in endosymbiont genomes due to selective processes involved in endosymbiont compensation and replacement in insects with minimal bacterial communities. The absence of essential gene pathways due to genomic erosion of long-term obligate endosymbionts, such as seen in *Carsonella*, means that S-endosymbionts are required to complement their role. This could also lead to P-endosymbiont replacement, exemplified by the loss of *Sulcia* from some Fulgoroidea (Auchenorrhyncha) lineages [21]; however, a loss of *Carsonella* has not been observed for any psyllid species. It could also lead to the replacement of one S-endosymbiont of a host species with another, and we have found evidence for this.

We interpret the presence of two or three clades of S-endosymbionts in individuals of psyllid species as a sign of incipient endosymbiont complementation or replacement, which warrants further examination. Multiple S-endosymbionts are common in whitefly, *Bemisia tabaci* (Hemiptera), and various combinations of the endosymbionts are found in its distinct biotypes (or species) whereby most S-endosymbionts are not fixed within host populations [9]. In *S. serrata*, *Sodalis* was more abundant than *Arsenophonus* in each of the three individuals; however, for *S. serrata*, only one population was sampled, and a more extensive screening of populations will reveal whether this bacterial community is indicative of this psyllid species across its distribution. In addition, whole genome sequencing of *Carsonella* and the two S-endosymbionts will be useful in assessing the genome characteristics of the endosymbionts and levels of metabolic complementarity. Competition between bacteriome-associated bacteria may be minimised if located in different bacteriocyte types [76]. In situ localisation of the bacterial cells to the host tissue is needed to reveal whether endosymbionts are either co-localised or compartmentalised, and this will be an essential step to further investigate the interaction dynamics between multiple bacterial species and the host. Other important information will be obtained by the comparative analysis of endosymbiont and host genomes.

Conceptually, symbiont complementation and replacement must begin with the opportunity for horizontal transmission of an endosymbiont into a new host, and this spillover acquisition [2] needs to be followed by establishment of the new endosymbiosis through reliable vertical transmission and host adaptation while some, if not most, of these new host-endosymbiont associations may be lost. The first contact may occur through introgression, interactions with parasitoids, parasites or predators, or via the sharing of a habitat [77, 78]. Psyllids have a large diversity of specialist parasitoids [50], with COI barcodes of parasitoids also detected in psyllid nymphs of this study, and they may play a role in transmission of endosymbionts across psyllid species. Similarly, the genetic relatedness of *Wolbachia* strains in highly divergent arthropod taxa demonstrates horizontal transmission. For *Rickettsia* endosymbionts, it has been demonstrated that they can be transmitted between whiteflies via phloem sap during feeding [79]. Some of the psyllid species in our study feed on related, but not identical host plant species, yet host plants could play a role as transmission platform. Based on our experimental design, it is not possible for us to conclude whether host plants play a role in horizontal transmission of psyllid endosymbionts; however, a previous study has not found any *Cardiaspina* S-endosymbionts in *Eucalyptus* leaves [3].

### Facultative endosymbionts as potential reproductive manipulators

*Wolbachia* bacteria are well-studied mostly facultative endosymbionts, which commonly induce reproductive manipulations of their hosts, such as cytoplasmic incompatibility, feminisation or male-killing in diplodiploid hosts [80]. *Wolbachia* was only found at high prevalence in *T. magnoliae* (3/3 individuals); however, it has also been found at high prevalence in *D. citri* [26] and is fixed in three *Mycopsylla* species [31, 81], but it is unknown whether *Wolbachia* manipulates their host’s reproduction in any of these psyllid species. Furthermore, *Wolbachia* can have fitness benefits for their host under times of nutrient stress [82] and has become an obligate mutualist in its association with bedbugs where it is situated in the bacteriome provisioning its host with B vitamins [83].

*Rickettsia* is a species of Rickettsiales that has been found frequently in whiteflies [9] and aphids. In aphids, the presence of *Rickettsia* suppresses the abundance of the P-endosymbiont *Buchnera* and confers a fitness cost on the host [84]. In our study, two *Rickettsia* sequences were detected at low prevalence but high titres within individuals: the first in three eucalypt psyllids—*C. fiscella*, *GB Cardiaspina sp*. and *C. corniculata*—and the second in *Allocasuarina*-associated *A. dobsoni. Cardiaspina fiscella* was the only species to harbour both *Wolbachia* and *Rickettsia*, but these bacteria did not co-occur in the same individual.

*Lariskella* was found at 100% prevalence in *C. maniformis* and *Creiis* sp. 1, both collected from *Eucalyptus tereticornis*. This endosymbiont has previously been detected in stinkbugs, fleas and ticks. In stinkbugs, *Lariskella* co-occurs with the P-endosymbiont *Schneideria*, in the bacteriome and ovaries, and is vertically transmitted to offspring [85] while in our study, it was found with *Sodalis* and Enterobacteriaceae S-endosymbionts. *Lariskella* is also closely related to *Wolbachia* and *Rickettsia*, and it has been thought that it manipulates host reproduction [85]. It is not yet known whether any of these Alphaproteobacteria manipulate psyllid reproduction; no sex ratio bias in psyllid populations has been recorded so far [33], and to the best of our knowledge, no reports of cytoplasmic incompatibility exist for psyllids.

### Low abundance and prevalence of transient gut bacteria, potential pathogens and the absence of chloroplast DNA

Only six of 191 specimens carried bacteria other than the previously discussed endosymbionts at greater than 1% relative abundance, and these bacteria may be considered transient microbial associations (i.e. *Serratia*, *Enterobacter*, *Escherichia*, *Erwinia*, *Pseudomonas* and *Staphylococcus*). Overall, these bacteria were detected at very low prevalence, despite the use of whole psyllid individuals, including the gut. *Spiroplasma* was found in one *G. brimblecombei* individual. Different species of *Spiroplasma* are arthropod-associated symbionts that can impact insect fitness and reproduction [86, 87], or they can be plant pathogenic [40] whereby any transmission of plant pathogenic *Spiroplasma* by psyllids is unknown. Therefore, it is more likely that the *Spiroplasma* found in *G. brimblecombei* impacts psyllid fitness rather than plants but this needs further investigation.

One factor that may have contributed to findings of low incidence and prevalence of transient bacteria is the large number of eucalypt-associated species included in this study while the other species were obtained from many host plant taxa. However, there was no overall significant difference in the microbiota of eucalypt-feeding psyllids and psyllids with other host plant associations. It has previously been suggested that antimicrobial effects of plant secondary compounds in eucalypt-feeders may impact their microbiota; however, based on our study, this appears not to be the case for psyllid microbiota. It is far more likely that the feeding mode of psyllids generally limits exposure to microbes [22] other than phloem-limited bacteria (including plant-pathogenic bacteria) that can be acquired through feeding on plant sap [40]. We have detected one psyllid species, *A. dobsoni*, which may act as a vector of *Phytoplasma* as a plant-pathogenic bacterium of *Allocasuarina*. Despite our relatively large sampling effort, we have not detected any other known psyllid-vectored pathogens such as *Liberibacter*; however, a novel *Liberibacter* isolate has recently been reported in some but not all *A. solanicola* individuals [88]. Perhaps, the potential of gall- and lerp-forming psyllids to act as pathogen vectors is reduced due to the sessile feeding mode of their nymphs that only develop on a single host plant individual, while nymphs of free-living psyllids can move between host plant individuals and therefore acquire pathogens from infected plants and transmit to uninfected plants. Furthermore, only a proportion of psyllid individuals within populations that vector plant pathogenic bacteria may actually be infected (but see [89]).

The complete absence of chloroplast reads in the 16S rRNA gene amplicon data also confirmed the exclusive plant sap diet of psyllids. In contrast, chloroplast 16S rRNA genes were detected in microbiota analyses of other plant sap-feeding insects such as thrips [90] and leaf beetles using the same experimental method (AAGH, JLM and MR, personal observation).

### Limitations due to primer bias

The amplicon primers chosen for this study were suitable for amplifying members of the Enterobacteriaceae, such as *Arsenophonus* and *Sodalis*, which are known to be present in many psyllid species [3] as well as *Wolbachia* and other Rickettsiales, which have been detected previously in psyllids and numerous insect species [9, 31]. The 16S rRNA gene amplicon sequencing primers had much lower sequence identity to *Carsonella* sequences from psyllids, and up to 29% sequence reads in some individuals were *Carsonella*, whereas in other individuals *Carsonella* was not detected at all, although it was subsequently found using *Carsonella*-specific primers. Previous amplicon sequencing studies have discussed this issue [81] and attempted to mitigate this bias by modifying the amplicon sequencing primers to ensure amplification of the known P-endosymbionts without compromising amplification of other microbial taxa [22]. However, the focus of our experiment was on S-endosymbionts and other bacteria, and therefore, amplicon sequencing primers that did not amplify *Carsonella* efficiently were ideal and adequately detected the dominance of Enterobacteriaceae as S-endosymbionts in psyllid microbiota. Another study using the same methodology and amplicon sequencing primer set demonstrated that these primers were efficient in amplifying plant pathogens such as *Liberibacter* and endosymbiont *Profftella* (Betaproteobacteria) from *Diaphorina* psyllids (JLM, personal observation). At the same time, our study also demonstrated the potential risk of amplification bias in amplicon sequencing approaches if not complemented by other approaches. For example, bacteria-specific qPCR using *Carsonella*-, *Arsenophonus*- and *Sodalis*-specific primers established that these bacteria were in high densities in host individuals.

Illumina sequencing is prone to substitution errors [51], and such errors can undoubtedly account for many of the SNPs that produce OTUs that are of low read count and present in few individuals, as well as in individuals that harbour a phylogenetically similar OTU with a much greater read count [22]. This serves to bloat the alpha diversity by splitting a single bacterial line into multiple OTUs, but in this study, WGS demonstrated for some hosts that the P- and S-endosymbionts are single clonal associations, as would be expected for vertically transmitted bacteria that pass through a bottleneck each host generation. Such inflation of OTU composition is significant for insects that harbour as few as two microbial associates.

## Conclusions

Psyllids are an excellent model system to investigate the evolutionary transition from facultative to obligate endosymbiosis of bacteria, endosymbiont complementation and replacement. All psyllids displayed a minimal community of a few core bacteria, with each species harbouring the P-endosymbiont *Carsonella* and mostly a single S-endosymbiont of the family Enterobacteriaceae or other Gammaproteobacteria in each individual of a species. Remarkably, all individuals of five psyllid species harboured two to three abundant S-endosymbionts that were found in all individuals, and these multipartite relationships should be further investigated in the context of P- and S-symbiont complementation and replacement as well as endosymbiont genome evolution. No psyllid individual was without an abundant S-endosymbiont despite previous reports for the absence of bacteria other than *Carsonella* in a few gall-forming psyllids. On the contrary, the diversity of microbial associations in gall formers was higher than that in free-living psyllids. Unexpectedly, diversity of S-endosymbionts in flush-feeding *C. eucalypti* and *G. brimblecombei* was greater in their native range than in the invasive range and invites investigation of the ecological and environmental factors that triggered the losses of S-endosymbionts from invasive populations while these S-endosymbionts appeared fixed in native populations.

## Additional files

Additional file 1: Sampling information for 191 specimens individually subjected to 16S rRNA gene amplicon sequencing, including number of reads generated by MiSeq amplicon sequencing before and after quality control, and following chimera removal (XLSX 48 kb).

Additional file 2: Primer sequences and PCR conditions (XLSX 11 kb).

Additional file 3: List of the nymphs collected for this study. Each nymph sample was subjected to COI amplification and sequencing. Samples with parasitoid DNA present were discarded. The list includes the number of nymphs from each species and site that were subject to DNA extraction, the number of those DNA extractions that were determined to be parasitised and the number of psyllid nymphs remaining that were included in the final 16S rRNA gene amplicon dataset (XLSX 12 kb).

Additional file 4: qPCR primer efficiency (XLSX 10 kb).

Additional file 5: Microbial OTU richness in 191 individuals of 25 psyllid species (summarised by species). Rarefaction curves were generated for each species from the mean number of observed OTUs (of 10 iterations) calculated from 40 evenly spread sampling depths to an upper limit of 2000 reads. Sampling was performed using alpha_rarefaction.py command within QIIME and the graph was plotted in R (PDF 31 kb).

Additional file 6: Alpha diversity metrics (XLSX 21 kb).

Additional file 7: OTU table for 191 samples. Original dataset was rarefied to 970 reads (the lowest number of reads in a sample) (XLSX 718 kb).

Additional file 8: Phylogenetic tree estimated using Bayesian inference. The 50 most abundant OTUs from the amplicon sequencing, plus *Carsonella* sequences either from amplicon or WGS datasets. Full-length or near full-length 16S rRNA gene sequences of eight S-endosymbionts were extracted from WGS datasets. Finally, 57 near full-length reference sequences of P- and S-endosymbionts of Hemiptera were retrieved from GenBank (PDF 13 kb).

Additional file 9: Normalised qPCR results (XLSX 14 kb).

Additional file 10: Beta diversity comparison of three distance matrices (unweighted UniFrac, weighted UniFrac and Bray-Curtis), with samples categorised by species, genus, ecological niche, feeding and host plant type (XLSX 9 kb).
