# Supplementary figures and images for: Symbionts in waiting: the dynamics of incipient endosymbiont complementation and replacement in minimal bacterial communities of psyllids
Source: Microbiome. 2017 Jun 6;5:58. doi: 10.1186/s40168-017-0276-4 (PMC5461708; doi:10.1186/s40168-017-0276-4)

# Rarefaction

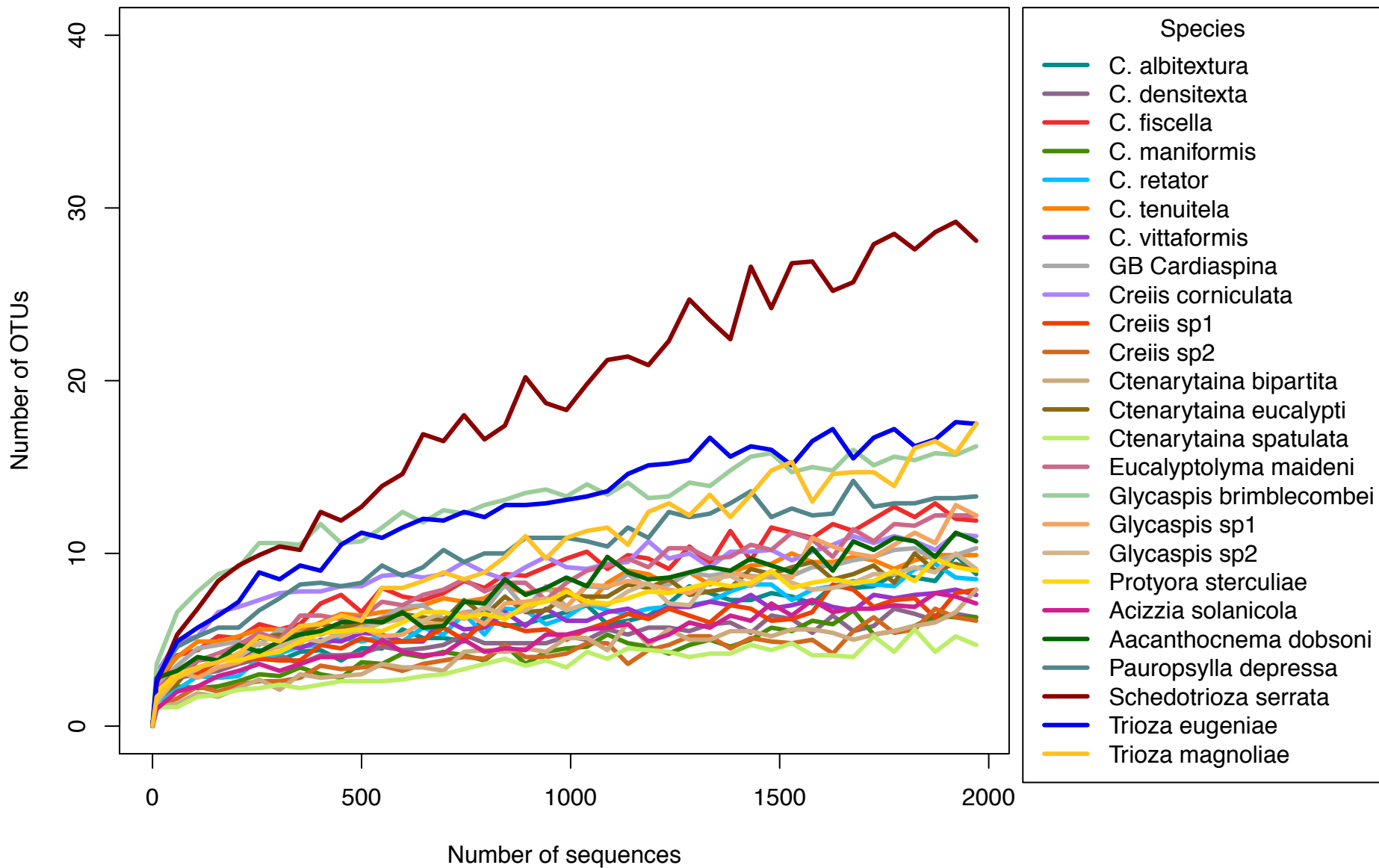

Supplement: Supplementary file 5 — Microbial OTU richness in 191 individuals of 25 psyllid species (summarised by species). Rarefaction curves were generated for each species from the mean number of observed OTUs (of 10 iterations) calculated from 40 evenly spread sampling depths to an upper limit of 2000 reads. Sampling was performed using alpha_rarefaction.py command within QIIME and the graph was plotted in R (PDF 31 kb). [file 40168_2017_276_MOESM5_ESM.pdf]

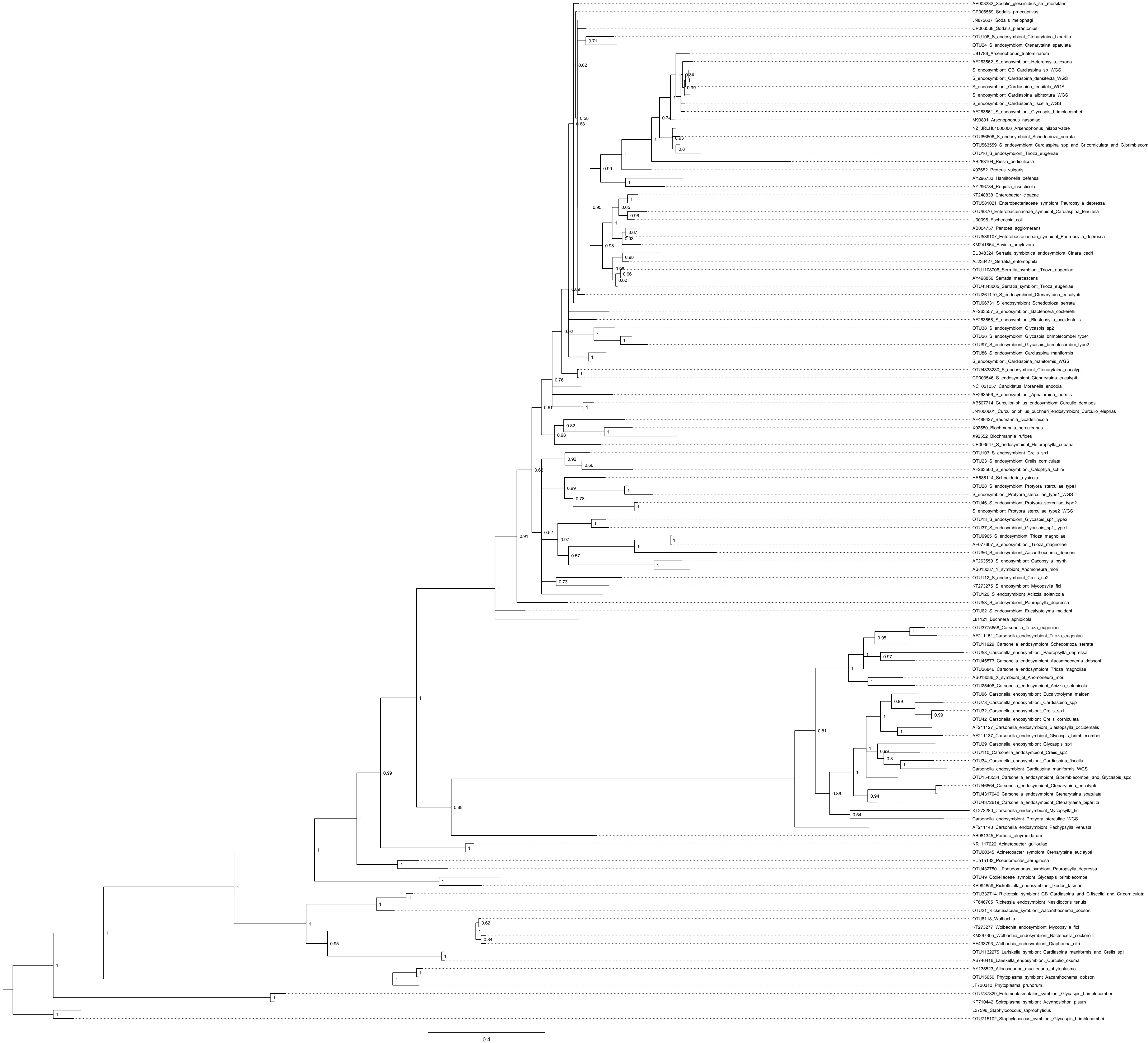

Supplement: Supplementary file 8 — Phylogenetic tree estimated using Bayesian inference. The 50 most abundant OTUs from the amplicon sequencing, plus Carsonella sequences either from amplicon or WGS datasets. Full-length or near full-length 16S rRNA gene sequences of eight S-endosymbionts were extracted from WGS datasets. Finally, 57 near full-length reference sequences of P- and S-endosymbionts of Hemiptera were retrieved from GenBank (PDF 13 kb). [file 40168_2017_276_MOESM8_ESM.pdf]
